# The Stress-Active Cell Division Protein ZapE Alters FtsZ Filament Architecture to Facilitate Division in *Escherichia coli*

**DOI:** 10.3389/fmicb.2021.733085

**Published:** 2021-09-27

**Authors:** Eric C. DiBiasio, Rebecca A. Dickinson, Catherine E. Trebino, Colby N. Ferreira, Josiah J. Morrison, Jodi L. Camberg

**Affiliations:** Department of Cell and Molecular Biology, The University of Rhode Island, Kingston, RI, United States

**Keywords:** cytokinesis, constriction, ATPase, AAA+, stress, Z-ring-associated protein

## Abstract

During pathogenic infections, bacterial cells experience environmental stress conditions, including low oxygen and thermal stress. Bacterial cells proliferate during infection and divide by a mechanism characterized by the assembly of a large cytoskeletal structure at the division site called the Z-ring. The major protein constituting the Z-ring is FtsZ, a tubulin homolog and GTPase that utilizes the nucleotide to assemble into dynamic polymers. In *Escherichia coli*, many cell division proteins interact with FtsZ and modulate Z-ring assembly, while others direct cell wall insertion and peptidoglycan remodeling. Here, we show that ZapE, an ATPase that accumulates during late constriction, directly interacts with FtsZ and phospholipids *in vitro*. In the presence of adenosine triphosphate (ATP), ZapE induces bundling of GTP-induced FtsZ polymers; however, ZapE also binds FtsZ in the absence of GTP. The ZapE mutant protein ZapE(K84A), which is defective for ATP hydrolysis, also interacts with FtsZ and induces FtsZ filament bundling. *In vivo*, cultures of *zapE* deletion cells contain a low percentage of filamentous cells, suggesting that they have a modest division defect; however, they are able to grow when exposed to stress, such as high temperature and limited oxygen. When combined with the chromosomal deletion of *minC*, which encodes an FtsZ disassembly factor, Δ*zapE* Δ*minC* cells experience growth delays that slow proliferation at high temperature and prevent recovery. This synthetic slow growth phenotype after exposure to stress suggests that ZapE may function to ensure proliferation during and after stress, and this is exacerbated when cells are also deleted for *minC.* Expression of either ZapE or ZapE(K84A) complements the aberrant growth phenotypes *in vivo* suggesting that the division-associated role of ZapE does not require ZapE ATP hydrolysis. These results support that ZapE is a stress-regulated cell division protein that interacts directly with FtsZ and phospholipids, promoting growth and division after exposure to environmental stress.

## Introduction

Proliferation via the highly conserved bacterial cell division pathway requires more than 30 different proteins acting in a well-coordinated manner (de Boer, 2010). While many of the individual cell division proteins involved have been characterized, knowledge of their functional interactions remains incomplete, including the interactions of late stage division proteins and those that direct peptidoglycan insertion or remodeling (Soderstrom et al., 2018, 2019). In *Escherichia coli* and most bacteria, cell division requires the highly conserved tubulin-like FtsZ protein (Bi and Lutkenhaus, 1991). FtsZ is a GTPase, and binding of GTP to FtsZ protomers leads to the formation of linear protofilaments arranged in a head-to-tail orientation (Du and Lutkenhaus, 2019). FtsZ protofilaments comprise the Z-ring at midcell during constriction and may act as a scaffold for assembly of the divisome (Soderstrom and Daley, 2017). A previous publication reported the presence of cell division protein ZapE in Gram-negative bacteria, including *E. coli*, and showed that ZapE was important for cell division at high temperature (42°C) and under anaerobic conditions (Marteyn et al., 2014). Moreover, ZapE was identified as essential in *Shigella flexneri* for colonization of the gastrointestinal tract (Marteyn et al., 2014).

FtsZ assembly into linear polymers and the dynamic exchange of FtsZ subunits at the Z-ring is regulated by many FtsZ-interacting proteins, including the “Z-ring-associated proteins” (Zaps). ZapA, -B, -C, and -D localize to the Z-ring early in the cell division pathway and regulate the development of the Z-ring (Schumacher et al., 2017). ZapE was characterized as a Z-associated protein, but ZapE is also a member of the AAA + ATPase family (ATPase associated with a variety of cellular activities) and contains both Walker A _78_(GGVGRGKT)_85_ and Walker B _141_(CFDEF)_145_ nucleotide-binding sites (Marteyn et al., 2014). The requirement for ZapE in *E. coli* grown under anaerobic and thermal stress conditions suggests that it may be a conditional cell division protein that is regulated at the transcriptional and/or translational levels in response to environmental conditions, or that it may become active *in vivo* during exposure to stress.

The mechanism by which ZapE impacts the cell division pathway through its direct interaction with FtsZ is largely unknown. ZapE is a late stage cell division protein that was reported to influence FtsZ polymerization *in vitro* by destabilizing FtsZ polymers in an adenosine triphosphate (ATP)-dependent manner (Marteyn et al., 2014). Another AAA + ATPase that has also been shown to influence FtsZ polymer assembly and destabilize FtsZ polymers is ClpX (Camberg et al., 2009; Viola et al., 2017). ClpX is a hexameric ATP-dependent unfoldase that, along with the serine protease ClpP, forms the two-component bacterial proteasome complex ClpXP. ClpXP processively degrades specific protein substrates, including FtsZ, after initiating ATP-dependent unfolding from the N- or C-terminus. During *E. coli* division, ClpXP degradation of polymerized FtsZ reduces polymer abundance and modifies the dynamic exchange of FtsZ subunits in the Z-ring by a polymer severing mechanism (Camberg et al., 2009, 2014; Viola et al., 2017). Most notably, however, ClpXP was previously reported to prevent the accumulation of intracellular FtsZ aggregates, which accumulate in *E. coli* grown under thermal stress conditions, including growth at 42°C (Tomoyasu et al., 2001; LaBreck et al., 2017). Other stress-induced protein chaperones, such as ClpB and HtpG, have also been implicated in modulating FtsZ aggregates and polymers (LaBreck et al., 2017; Balasubramanian et al., 2019).

To gain mechanistic insight into the functional roles and interactions of ZapE in *E. coli*, we monitored biochemical activities and direct interactions with polymerized and non-polymerized FtsZ *in vitro*. Our results clearly demonstrate a direct interaction between the two proteins and also show that ZapE can recruit a chimeric FtsZ protein to phospholipid (PL) vesicles. ZapE bundles FtsZ polymers in the presence of ATP, which is in contrast to disassembly activity that was previously reported (Marteyn et al., 2014). Consistent with the previous report, we also show that deletion of *zapE* induces cell filamentation in a small proportion of cells. Interestingly, we also show that deletion of *zapE* slows overall growth of a *minC* deletion strain, and this is complemented by reinserting wild-type *zapE* or *zapE(K84A)* back into the native locus. MinC is an FtsZ polymerization inhibitor that prevents division near the cell poles (Hu et al., 1999; Hu and Lutkenhaus, 2000; Dajkovic et al., 2008). Together, our results support that ZapE is critical for division under thermal and oxygen stress and may function to organize and maintain active populations of FtsZ *in vivo* to facilitate division.

## Materials and Methods

### Bacterial Strains, Plasmids, and Growth Conditions

*Escherichia coli* strains, listed in Table 1, were grown in LB (Lennox), Luria–Bertani broth with NaCl 5 g/L, media and/or LB (no salt) media and agar, plus ampicillin (100 μg ml^–1^), or kanamycin (50 μg ml^–1^), where indicated. Gene deletions were constructed by Lambda red recombination as described (Datsenko and Wanner, 2000), and chromosomal in-frame gene replacements with *zapE* or *zapE(K84A)* were constructed as described (LaBreck et al., 2019). Culture densities were monitored and measured by OD_600_. For protein purification, *zapE* and *zapE*(K84A) expression plasmids were constructed by restriction cloning (*Nde*I, *Hin*dIII) and site-directed mutagenesis into pET-28a expression vector with an N-terminal 6 × -histidine tag.

**TABLE 1:** *E. coli* strains and plasmids used for genetic analyses and constructions in this study.

*E. coli* strain or plasmid	Relevant genotype description	Source, reference or construction
Strains		
MG1655	*LAM-rph-1*	Blattner et al., 1997
BL21 (λde3)	F-*ompT gal dcm lon hsdSB(rB- mB-)* λ(de3[*lacI lacUV5-T7 gene 1 ind1 sam7 nin5*])	EMD Millipore, United States
ED0011	MG1655 Δ*zapE*:*parE-kan*	MG1655; λred
JC0232	MG1655 Δ*minC*:*frt*	Camberg et al., 2011
ED0024	MG1655 Δ*minC*:*frt*,Δ*zapE*: *parE-kan*	MG1655 Δ*minC*:*frt*; λred
ED0118	MG1655 Δ*minC*:*frt*Δ*zapE*:*zapE*	MG1655 Δ*minC*:*frt*Δ*zapE*: *parE-kan*; λred
ED0131	MG1655Δ*minC*:*frt*Δ*zapE*:*zapE(K84A)*	MG1655 Δ*minC*:*frt*Δ*zapE*: *parE-kan*; λred
**Plasmids**		
pKD46	*Amp*, *recombinase*	Datsenko and Wanner, 2000
pKD267	*parE-kan, tet*	LaBreck et al., 2019
pET-H_6_-ZapE	*kan*, *His_6_-zapE*	This study
pET-ZapE-H_6_	*kan*, *zapE-His_6_*	This study
pET-H_6_-ZapE(K84A)	*kan*, *His_6_-zapE(K84A)*	This study
pET-Gfp-FtsZ	*kan*, *His_6_-Gfp-ftsZ*	Viola et al., 2017
pET-FtsZ	*kan*, FtsZ	Camberg et al., 2009

### Protein Expression and Purification

Vectors containing *zapE* and *zapE(K84A)* with an N-terminal 6 × -histidine tag were transformed and overexpressed in *E. coli* BL21 (λde3). Cell cultures were grown to an OD_600_ of 0.8 and induced with IPTG (0.5 mM) for 4 h at 30°C. Cultures were centrifuged, and pellets were lysed by French press in 25 mM Tris (pH 7.5) buffer containing 0.1 mM TCEP, 10 mM MgCl_2_, 150 mM KCl, and 10% glycerol. Soluble cell extracts were collected by centrifugation at 35,000 × *g* for 30 min, applied to a cobalt column, and eluted with an imidazole gradient (0 mM to 150 mM). Collected protein was fractionated on a sephadex G25 column to remove imidazole and small contaminants. Native FtsZ was expressed in *E. coli* BL21 (λde3) and purified as described (Camberg et al., 2009). ZapE containing a C-terminal 6 × -histidine tag was purified as described above. Histidine-tagged Gfp-FtsZ, which contains green fluorescent protein (Gfp) fused to the N-terminus of FtsZ, was expressed in *E. coli* BL21 (λde3) and purified as described (Viola et al., 2017). Where indicated, size exclusion chromatography was performed on purified H_6_-ZapE. Briefly, H_6_-ZapE was incubated at room temperature with or without ATP (5 mM) for 5 min and then passed through a Sephacryl S-200 (25 ml) column with 25 mM Tris (pH 7.5) buffer containing 0.1 mM TCEP, 10mM MgCl_2_, 150 mM KCl, and 10% glycerol, and, where indicated, equilibrated with ATP (0.5 mM). Eluting fractions (250 μl) were collected (0.5 ml min^–1^) and analyzed by the Bradford protein assay and the proteins were visualized by SDS-PAGE and Coomassie staining.

### Nucleotide Hydrolysis Assays

Hydrolysis of ATP or GTP, where indicated, by ZapE or FtsZ in 50 mM HEPES buffer, pH 7.5, containing 150 mM KCl and 20 mM MgCl_2_ in the absence or presence of SUVs (250 μg ml^–1^), where indicated, was monitored by measuring the release of inorganic phosphate with time using the Biomol Green phosphate detection reagent (Enzo Life Sciences) at 23°C as described (Camberg et al., 2014). Free phosphate was quantitated by comparison with a phosphate standard curve. Where indicated, ATP concentration was titrated in hydrolysis reactions, and the results were analyzed in GraphPad Prism (version 8) by fitting data to the Michaelis–Menten equation.

### FtsZ Sedimentation Assays

FtsZ polymers were assembled by incubating FtsZ (6 μM) in 50 mM HEPES buffer, pH 7.5, containing 150 mM KCl, 20 mM MgCl_2_ with GTP (2 mM) in the absence and presence of ZapE or ZapE(K84A) (8 μM) with or without ATP or ATPγS (4 mM), where indicated, at 23°C for 10 min and collected by centrifugation for 20 min at 250,000 × *g*, Supernatants and pellets were resuspended in equivalent volumes of LDS sample buffer (Life Technologies) and analyzed by SDS-PAGE and Coomassie staining.

### Light Scattering Assays

Light scattering (90° angle) was performed in 50 mM MES (pH 6.5) buffer containing 100 mM KCl and 10 mM MgCl_2_. Where indicated, reaction mixtures (80 μl) containing ZapE (0 to 8 μM) or ZapE(K84A) (4 μM), with or without FtsZ (8 μM), were monitored at 23°C using an Agilent Eclipse fluorescence spectrophotometer with both excitation and emission wavelengths set to 450 nm with 5 nm slit widths, as described (Conti et al., 2018). Protein reactions were incubated at room temperature for 5 min, and where indicated, with ATP (4 mM) or ATPγS (2 mM). Baseline readings were collected for 5 min, GTP (1.5 mM) was added, and light scattering was measured at 23°C for up to 120 min, where indicated.

### Binding and Retention Assay

Binding of ZapE to FtsZ was assayed by dot blot. Briefly, bovine serum albumin (BSA) (10 μM), ZapE (10 μM), and FtsZ (10 μM) alone or with GDP (2 mM) or GTP (2 mM) were spotted (3 μl) onto a nitrocellulose membrane. The membrane was blocked with BSA (2%) in tris-buffered saline (TBS) then incubated with ZapE (8 μM) and ATP (4 mM) in 50 mM HEPES (pH 7.5) buffer containing 150 mM KCl and 20 mM MgCl_2_ at 23°C for 1 h to allow binding. After washing with TBS containing Tween-20 (0.01%), the membrane was probed with rabbit polyclonal antisera (anti-ZapE) and then goat anti-rabbit antibody coupled to horseradish peroxidase. The membrane was developed by chemiluminescence, and spot density was analyzed by NIH ImageJ. Anti-ZapE antiserum was generated in rabbits using purified H_6_-ZapE (ThermoFisher).

Filter retention assays were performed to detect interactions between fluorescent Gfp-FtsZ and ZapE *in vitro*. ZapE (5 μM), ZapE(K84A) (5 μM), and/or Gfp-FtsZ (10 μM) reaction mixtures (40 μl), in 50 mM MES (pH 6.5) buffer containing 100 mM KCl and 10 mM MgCl_2_, were incubated with ATP (4 mM) and, where indicated, GTP (2 mM) at 23°C for 10 min. Reactions were filtered through 100 kDa Nanosep polyethersulfone filters by centrifugation at 16,900 × *g* for 20 min. Protein present in filtrate and retentate were analyzed by SDS-PAGE and Coomassie staining, and densitometry was performed using NIH ImageJ.

### Phospholipid Recruitment Assays

Phospholipid recruitment assays with ZapE were performed by incubating ZapE (1 μM) or ZapE K84A (1 μM) together with small unilamellar vesicles (SUVs) (500 μg ml^–1^) for 5 min at 23°C in the presence or absence of ATP (4 mM), where indicated in 50 mM HEPES (pH 7.5) reaction buffer containing 150 mM KCl and 20 mM MgCl_2_. Phospholipid recruitment assays with ZapE and Gfp-FtsZ (3 μM) were performed by incubating Gfp-FtsZ with GTP (4 mM), where indicated, in reaction buffer for 2 min and then added to a reaction containing ZapE (1 μM) pre-assembled with SUVs (500 μg ml^–1^) and ATP (4 mM) and then incubated for an additional 2 min at room temperature. Phospholipid vesicles and associated proteins were collected by low-speed centrifugation at 21,000 × *g* for 15 min. Supernatants and pellets were analyzed by SDS-PAGE and Coomassie staining. Percent of protein in the pellet fraction was determined by densitometry using NIH ImageJ.

### Microscopy

MG1655, MG1655 Δ*zapE*, MG1655 Δ*zapE*:*zapE*, *and* MG1655 Δ*zapE*:*zapE(K84A)* strains were grown overnight in LB (Lennox) media at 30°C. The next day, cultures were diluted into fresh LB (Lennox) medium standardized to OD_600_ of 0.05, grown for 3 h at 35°C, and then the final OD_600_ was measured. Cells were then harvested by centrifugation at 3,000 × *g* for 5 min, resuspended in 100 μl of 1 × PBS with EDTA (1 mM), and fixed to glass slides with poly-_*L*_-lysine-coated coverslips or, where indicated, applied to glass slides with agarose gel pads (5%, wt/vol) containing M9 minimal medium supplemented with 0.4% glucose. Cells were imaged by DIC microscopy using a Zeiss AxioCam HRc high-resolution camera, processed using Adobe photoshop CS6, and measured using ImageJ software.

For electron microscopy imaging, reactions containing buffer (50 mM MES pH 6.5, 100 mM KCl, 10 mM MgCl_2_), ZapE (8 μM), ZapE (K84A) (8 μM), FtsZ (8 μM), and ATP (4 mM), where indicated, were incubated for 5 min at 23°C, then GTP (1.5 mM), was added to induce complex formation, where indicated, and reactions were incubated for an additional 15 min at room temperature. Samples were applied to a 300-mesh carbon/formvar coated grid, fixed with glutaraldehyde (2.5%), and negatively stained with uranyl acetate (2%). Samples were visualized at × 10,000— × 25,000 direct magnification by transmission electron microscopy using a JEM-2100 80 Kev instrument.

### Colony Growth Assays

MG1655, MG1655 Δ*zapE*, MG1655 Δ*minC*, MG1655 Δ*minC*Δ*zapE*, MG1655 Δ*minC*Δ*zapE*:*zapE*, *and* MG1655 Δ*minC*Δ*zapE*:*zapE(K84A)* strains were grown overnight in LB (Lennox) medium at 30°C. The next day, cultures were diluted into fresh LB (no salt) or LB (Lennox) NaCl 5 g/L medium, where indicated, standardized to an OD_600_ of 0.05 and grown for 3 h under mild heat shock conditions in static, closed culture at 42°C, or aerobically at 37°C and shaking. After the allotted time, growth was measured by OD_600_, and cultures were diluted (2-log) into LB (no salt) medium. Dilutions were spot plated (5 μl) onto Lennox agar LB (no salt) agar, and plates were incubated at room temperature. Images of colony growth were collected, and biomass was quantified in ImageJ software to determine spot density in the zone of growth.

### Western Blotting

MG1655, MG1655 Δ*zapE*, MG1655 Δ*minC*, MG1655 Δ*minC*Δ*zapE*, MG1655 Δ*minC*Δ*zapE*:*zapE*, *and* MG1655 Δ*minC*Δ*zapE*:*zapE(K84A)* strains were grown overnight in LB (Lennox) media at 30°C. The next day, cultures were diluted into fresh LB (no salt) to an OD_600_ of 0.05 and grown for 3 h under mild heat shock conditions in static, closed cultures at 42°C, or aerobically at 37°C with shaking. Cells were harvested, normalized to total number of cells for SDS-PAGE, and then cell extracts were analyzed by Western blotting. Membranes were blocked with BSA (2%) in TBS containing Tween-20 (0.005%), and ZapE was detected with rabbit polyclonal antisera raised against ZapE followed by goat anti-rabbit antibody coupled to horseradish peroxidase. Membranes were developed using chemiluminescence.

## Results

### ZapE Interacts With FtsZ *In vitro* and Bundles FtsZ Polymers in an ATP-Dependent Manner

**FIGURE 1:**
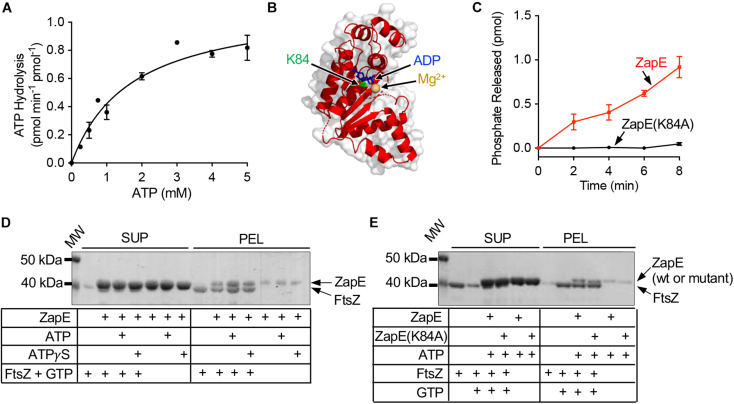
ZapE hydrolyzes ATP and co-pellets with FtsZ polymers *in vitro*. **(A)** ZapE (12 μM) hydrolysis rates were measured by monitoring the release of phosphate with increasing ATP concentrations (0 to 5 mM) as described in *Materials and methods* section. Data shown are the average of at least three replicates represented as mean ± SEM. **(B)** Structural model of *E. coli* ZapE residues (51–275) modeled onto DnaA (pdb 2Z4R) (Ozaki et al., 2008) with nucleotide shown in blue (ADP). Lys 84 is shown as green Corey–Pauling–Koltun model. **(C)** Hydrolysis of ATP (4 mM) by ZapE (6 μM) and ZapE(K84A) (6 μM) was monitored with time (0, 2.0, 4.0, 6.0, and 8.0 min) and phosphate released (pmol) was reported. Data shown are the average of at least three replicates represented as mean ± SEM. **(D)** FtsZ (6 μM) polymers were assembled with GTP (2 mM) in the absence and presence of ZapE (8 μM) alone and with ATP or ATPγS (4 mM). FtsZ polymers were collected by ultracentrifugation, as described in *Materials and methods* section, and contents of supernatants and pellets were analyzed by SDS-PAGE to detect ZapE. Data shown are representative of at least three replicates. **(E)** FtsZ (6 μM) was incubated without GTP, where indicated, or assembled into polymers with GTP (2 mM) in the absence and presence of ZapE (8 μM) or ZapE(K84A) (6 μM) with ATP (4 mM). FtsZ polymers were collected by ultracentrifugation, as described in *Materials and methods* section, and contents of supernatants and pellets were analyzed by SDS-PAGE to detect ZapE. Data shown are representative of at least three replicates.

*Escherichia coli* ZapE was previously reported to engage FtsZ *in vitro* and localize to the Z-ring *in vivo* (Marteyn et al., 2014). To biochemically characterize the enzymatic activity and interaction between ZapE and FtsZ further, we purified ZapE as an N-terminal six histidine fusion protein by metal affinity chromatography and then assayed ZapE for ATP hydrolysis activity and a direct interaction with FtsZ (Figure 1A and Supplementary Figure 1A). First, we titrated substrate (ATP) concentration from 0 to 5 mM in reactions containing ZapE (12 μM) to determine the maximal reaction velocity (V_*max*_). The reaction velocity plateaued near ∼1 pmol Pi min^–1^ pmol^–1^. Curve fitting to the Michaelis–Menten equation calculated a V_*max*_ of 1.1 ± 0.09 pmol Pi min^–1^ pmol^–1^ with a K_*m*_ of 1.7 ± 0.36 mM, under the conditions tested (Figure 1A). To determine the oligomeric state of ZapE, we fractionated ZapE by size exclusion chromatography in the absence and presence of ATP, and calculated the size of ZapE to be ∼40 kDa under both conditions based on the elution volume of the peak fraction (Supplementary Figures 1B,C), which is consistent with the calculated molecular mass of 44,583 Da. However, we also observed that the elution peak was broader with an early eluting shoulder in the presence of ATP, which could suggest a minor population of dimers in equilibrium with the predominant monomer fraction (Supplementary Figures 1B,C). We detected no apparent positive cooperativity upon titrating protein or substrate concentration in ATP hydrolysis assays (Figure 1A and Supplementary Figure 1D), which is consistent with ZapE functioning as a monomer in these reactions. Notably, we also constructed a C-terminally tagged six histidine fusion protein, but it hydrolyzed ATP at an approximately 65% slower rate (0.31 ± 0.02 pmol Pi min^–1^ pmol^–1^) than ZapE with the N-terminal histidine tag, suggesting that the addition of a tag sequence to the C-terminus of ZapE may interfere with ATPase activity (Supplementary Figure 1E). A detailed structural model of the complete ZapE protein is unavailable; therefore, to analyze ZapE functional regions in further detail, we modeled ZapE onto DnaA, which has 44% similarity across a region of ZapE (amino acids 74 through 181) that overlaps the Walker A motif (Figure 1B) (Ozaki et al., 2008). The substitution mutation K84A in the Walker A motif of ZapE was previously reported to impair ATP hydrolysis *in vitro* by thin layer chromatography; therefore, we purified ZapE(K84A) as an N-terminal histidine fusion protein to quantitatively measure ATP hydrolysis. We observed no phosphate released in reactions containing ZapE(K84A) (6 μM) and ATP under the conditions tested, in contrast to wild-type ZapE (Figure 1C). Together, these results demonstrate that ZapE is an ATPase and substitution of Lys 84 with Ala prevents activity, consistent with a previous report (Marteyn et al., 2014).

ZapE was implicated in regulating FtsZ polymerization in qualitative fluorescence-based microscopy assays *in vitro* (Marteyn et al., 2014), suggesting that there is a direct interaction with polymerized FtsZ. To detect the interaction, we first tested if ZapE co-pellets with FtsZ polymers assembled with GTP. We incubated ZapE with FtsZ in the presence of GTP and collected FtsZ polymers by ultracentrifugation. After fractionating supernatants and pellets, we analyzed both fractions by SDS-PAGE for the relative amounts of FtsZ and ZapE. We observed that FtsZ, incubated with GTP to promote polymerization, fractionates with the pellet, consistent with the assembly of GTP-dependent polymers (Figure 1D). When polymerized FtsZ was incubated with ZapE, pellet fractions also contained ZapE along with FtsZ. In a control reaction without FtsZ, only a small amount of ZapE was detected in the centrifugation pellet. Together, these results suggest that ZapE interacts with FtsZ polymers. Both protein bands migrated very closely by SDS-PAGE due to their size similarity complicating any quantitation by densitometry. When ATP or ATPγS was included in the reaction, the amount of ZapE co-pelleting with FtsZ and GTP increased, relative to reactions performed in the absence of ATP. These results show that ZapE co-pellets with FtsZ and GTP, and that ATP increases the amount of ZapE in the pellet with FtsZ. Next, as a control, we confirmed that FtsZ sediments only in the presence of GTP and does not fractionate with the pellet when GTP is omitted, consistent with specific GTP-dependent polymerization of FtsZ (Figure 1E). Finally, we tested the ZapE mutant protein ZapE(K84A), which is defective for ATP hydrolysis, and observed that it also co-pellets with FtsZ and GTP (Figure 1E).

**FIGURE 2:**
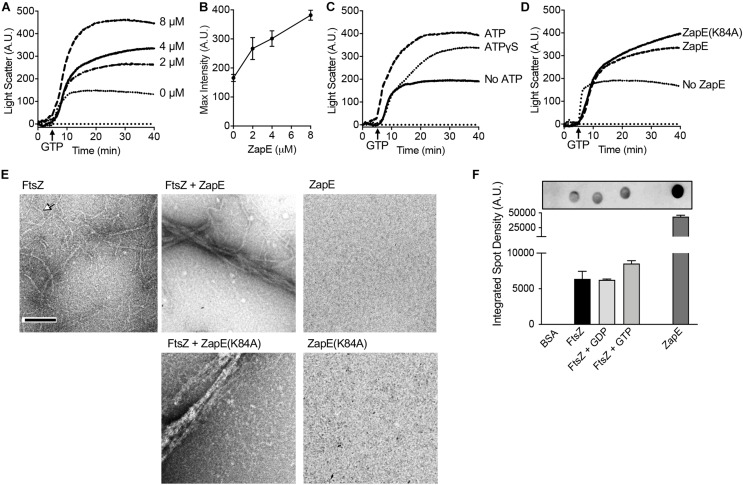
ZapE promotes FtsZ polymer bundling. **(A)** GTP-dependent assembly of complexes containing FtsZ was monitored by 90° light scattering, as described in *Materials and methods* section. FtsZ (8 μM) was incubated with increasing concentration of ZapE (0 to 8 μM) in the presence of ATP (4 mM). After collecting a baseline for 5 min, GTP (1.5 mM) was added to stimulate FtsZ polymerization and light scatter was monitored over time. **(B)** Maximum intensity (A.U.) values after addition of GTP in **(A)** for light scattering reactions containing FtsZ (8.0 μM) and increasing concentrations of ZapE titration (0, 2.0, 4.0, and 8.0 μM) and ATP (4 mM). Data shown are the average of at least three replicates represented as mean ± SEM. **(C)** Light scattering assay showing reactions containing FtsZ (8.0 μM) and ZapE (8.0 μM) in the absence or presence of ATP (4 mM) or ATPɣS (2 mM). Data shown are representative of at least three replicates. **(D)** Light scattering assay showing reactions containing FtsZ (8 μM), assembled with GTP (1.5 mM) in the absence and presence of ZapE (4 μM) or ZapE(K84A) (4 μM) with ATP (4 mM). Data shown are representative of at least three replicates. **(E)** ZapE (8 μM) or ZapE(K84A) (8 μM) were incubated with ATP (4 mM) and, where indicated, FtsZ (8 μM) and GTP (1.5 mM). Reactions were incubated for 15 min at 23°C and then visualized by transmission electron microscopy (TEM) as described in *Materials and methods* section. Size bars are 200 nm. Arrow indicates an FtsZ polymer. **(F)** Binding of ZapE to FtsZ was assayed by dot blot. Bovine serum albumin (BSA) (10 μM), ZapE (10 μM), and FtsZ (10 μM) alone or with GDP (2 mM) or GTP (2 mM) were spotted (3 μl) onto a nitrocellulose membrane and then membranes were blocked, incubated with ZapE (8 μM) and ATP (4 mM) to allow binding, and then probed with antibodies to ZapE. Spot densities were quantified by NIH ImageJ. Data shown are the average of at least three replicates represented as mean ± SEM.

To directly observe the effects of ZapE on FtsZ polymerization in real time *in vitro*, we used 90° light scattering to monitor GTP-dependent polymerization. First, we pre-incubated FtsZ (8 μM) with ZapE (0 to –8 μM) in the presence of ATP (4 mM) for 5 min, added GTP (1.5 mM) to induce polymerization, and monitored the change in light scatter with time. We observed that as ZapE concentration increases, the light scattered and maximum intensity recorded also increased, with a greater than twofold increase in maximum light scatter comparing ZapE (0 μM) and ZapE (8 μM) (Figures 2A,B). The increase in light scatter by FtsZ has been reported to correspond to the accumulation of FtsZ polymers after addition of GTP (Mukherjee and Lutkenhaus, 1994, 1999; Camberg et al., 2014). In a control assay, no increase in light scatter was detected for ZapE alone after addition of GTP (Supplementary Figure 2A). We also performed a longer time course of the FtsZ polymer assembly reaction to determine if polymers persisted or rapidly disassembled with ZapE. We observed that the kinetics of disassembly, which can be observed during minutes 40 through 90, appear similar with and without ZapE, although large complexes did persist longer with ZapE (Supplementary Figure 2B).

To determine if ZapE slows the GTPase activity of FtsZ, like other *E. coli* proteins reported to bundle FtsZ polymers (Gueiros-Filho and Losick, 2002; Low et al., 2004; Small et al., 2007; Hale et al., 2011; Durand-Heredia et al., 2012), we measured the rate of GTP hydrolysis in reactions containing FtsZ (6 μM) in the absence and presence of ZapE (12 μM). The GTP hydrolysis rate of FtsZ was determined to be 1.00 ± 0.28 pmol P_*i*_ min^–1^ pmol^–1^ under the conditions tested, and the rate did not significantly change in the presence of ZapE 1.13 ± 0.31 pmol P_*i*_ min^–1^ pmol^–1^ (Supplementary Figure 2C). Additionally, ZapE is unable to use GTP as a substrate under the conditions tested (data not shown). We also tested if FtsZ modifies ZapE ATPase activity but detected no change in the rate of ATP hydrolysis by ZapE when FtsZ was included in the reaction (Supplementary Figure 2D). Together, these results show that addition of ZapE to FtsZ polymerization reactions increases the scatter of protein complexes containing ZapE and FtsZ, suggesting the ZapE may bundle or functionally crosslink FtsZ polymers. However, we did not detect that ZapE can modify the GTP turnover by FtsZ under the conditions tested.

Next, to determine if ATP is necessary for the observed increase in light scatter stimulated by ZapE in FtsZ polymerization reactions, we repeated the assay without ATP. We did not detect ZapE-induced stimulation of GTP-dependent light scatter when ATP was omitted from FtsZ polymerization reactions containing ZapE (Figure 2C). Next, we tested ATPγS (2 mM) in ZapE and FtsZ 90° light scattering assays (Figure 2C) and observed a modest increase in light scatter compared with the increase in the absence of ATP (Figure 2C). Finally, we also tested if ZapE(K84A) stimulates FtsZ light scatter in GTP-dependent polymerization assays and observed an increase similar to wild-type ZapE with ATP (Figure 2D). These results suggest that nucleotide binding by ZapE, but not hydrolysis, is sufficient to induce assembly of large complexes with FtsZ polymers. To determine if the large complexes that we detected are bundled FtsZ polymers, we performed transmission electron microscopy (TEM) and negative staining of FtsZ polymers assembled with GTP in the absence and presence of ZapE and ATP, the condition that increases light scatter (Figure 2E). We observed that without ZapE, FtsZ polymers were long and thin, and many polymers detected appeared to be single-stranded with some thicker filaments present (Figure 2E). In contrast, in the reaction containing FtsZ polymers incubated with ZapE, polymers were grouped together as large bundles of approximately 100 nm in width, and no polymers or bundles were observed by ZapE without FtsZ (Figure 2E). Bundles were also observed when FtsZ polymers were incubated with ZapE(K84A), which is consistent with light scatter assays (Figures 2D,E).

Polymerization and light scattering assays both indicated that ZapE directly binds to FtsZ polymers; however, to determine if ZapE also binds to FtsZ in the absence of GTP, we performed dot blot binding assays by applying purified FtsZ to a nitrocellulose membrane in the presence of various nucleotides and then monitored ZapE recruitment. Briefly, BSA (10 μM), ZapE (10 μM), FtsZ (10 μM), FtsZ (10 μM) with GDP (2 mM), and FtsZ (10 μM) with GTP (2 mM) were applied as a spot to a nitrocellulose membrane and then blocked with BSA. A solution of ZapE (8 μM) with ATP (4 mM) was incubated with the membrane to allow for potential binding. Binding was then probed with a ZapE antibody. Our results show that ZapE binds FtsZ similarly under all conditions tested and that ZapE does not bind to the control spot containing BSA (Figure 2F). These results suggest that ZapE binds to both the unpolymerized and polymerized states of FtsZ. To more quantitatively compare ZapE binding to FtsZ and the absence and presence of GTP, we collected FtsZ–ZapE complexes by ultrafiltration on a polyethersulfone filter with a 100-kDa exclusion limit. To analyze retained proteins and clearly differentiate ZapE from FtsZ by SDS-PAGE, since they migrate similarly due to their close size, we utilized the chimeric fusion protein Gfp–FtsZ. Gfp–FtsZ, which has a molecular weight of 68 kDa, was previously shown to polymerize, hydrolyze GTP, and behave similarly to wild type FtsZ in *in vitro* assays and assemble into a Z-ring *in vivo* (Viola et al., 2017). In the presence of Gfp–FtsZ (10 μM) and ATP (4 mM), 63.4% of the total ZapE in the reaction (5 μM) was retained in complex with Gfp–FtsZ, whereas only 33.4% of ZapE was retained on the filter when Gfp–FtsZ was omitted, suggesting that ZapE forms a complex with FtsZ in the absence of nucleotide (Supplementary Figure 2E). The amount of ZapE that was retained did not change in the presence of GTP, which promotes FtsZ polymerization, suggesting that ZapE binds similarly to non-polymerized and polymerized Gfp–FtsZ (Supplementary Figure 2E). Finally, to determine if ATP hydrolysis is required for complex formation between ZapE and FtsZ, we performed ultrafiltration assays with ZapE(K84A) (5 μM), co-incubated with FtsZ (10 μM) and ATP (4 mM), and also observed that Gfp–FtsZ retained ZapE(K84A) at a higher value (80.2%), in comparison with ZapE(K84A) alone 50.3% (Supplementary Figure 2F), suggesting that ZapE nucleotide hydrolysis is not required for Gfp–FtsZ binding.

### Phospholipid Binding and Recruitment of FtsZ

**FIGURE 3:**
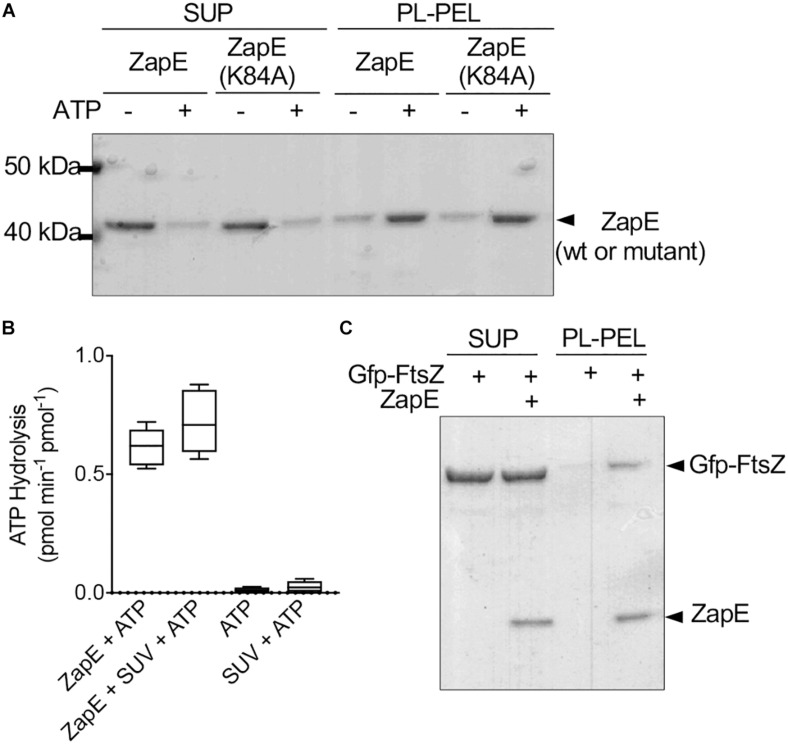
ZapE recruits FtsZ to phospholipid (PL) vesicles *in vitro*. **(A)** ZapE (1 μM) or, where indicated, ZapE(K84A) (1 μM), was incubated with *E. coli* PL vesicles (500 μg ml^–1^) in the absence or presence of ATP (4 mM). PL-associated protein was collected by low-speed centrifugation. Supernatants and pellets were analyzed by SDS-PAGE. Data shown are representative of at least three replicates. **(B)** ATP hydrolysis assays were performed in the presence of PL vesicles in reactions containing ZapE (6 μM), ATP (4 mM), and PL vesicles (250 μg ml^–1^). Data shown are the average of at least three replicates represented as mean ± SEM. **(C)** Gfp-FtsZ (3 μM) was incubated with GTP (4 mM) and added to PL vesicles (500 μg ml^–1^) with and without ZapE (1 μM) and ATP (4 mM). PL-associated protein was collected by low-speed centrifugation. Supernatants and pellets were analyzed by SDS-PAGE. Data shown are representatives of at least three replicates.

In addition to FtsZ, ZapE was reported to be present in complexes with FtsQ, FtsI, FtsL, and FtsN, which are all late cell division proteins and also integral membrane proteins (Marteyn et al., 2014). Therefore, we tested if ZapE could bind to *E. coli* PL directly in a phospholipid recruitment assay (Figure 3A). ZapE was incubated with PL vesicles (500 μg ml^–1^) in the absence and presence of ATP (4 mM), and then supernatant and PL-pellet fractions were collected by low-speed centrifugation and analyzed by SDS-PAGE (Figure 3A). We observed that in the presence of ATP, 76% of ZapE fractionated with PLs; however, only 23.6% of ZapE was recruited to the PL pellet in the absence of ATP, suggesting that PL binding is modulated by ATP. In the absence of SUVs, ZapE remained in the supernatant with and without ATP (Supplementary Figure 2G). ZapE(K84A) showed a similar fractionation pattern where 80.3% of ZapE(K84A) was recruited to the PL pellet in the presence of ATP (Figure 3A). To determine if the interaction was disrupted by increasing ionic strength, we repeated the PL recruitment assay with ZapE and ZapE(K84A) in the presence of ATP and 400 mM NaCl. We observed that ATP-dependent association with PLs was tolerant to 400 mM NaCl (Supplementary Figure 2H), but was disrupted at higher NaCl concentrations (data not shown). This suggests that the binding detected is likely due to an electrostatic interaction at the PL surface (i.e., phosphate head group). Next, we tested if the addition of PLs to ZapE modifies the ATP hydrolysis activity in ATPase activity assays; however, we observed no change in the rate of ATP hydrolysis by ZapE when PLs were included in the reaction (Figure 3B). Finally, to determine if ZapE recruits FtsZ to PLs, we incubated Gfp-FtsZ with GTP and PLs in the absence and presence of ZapE and ATP (Figure 3C). We observed that 21.6% of the total Gfp-FtsZ included in the reaction fractionated with PLs when ZapE was also present, but only 6% of the total Gfp-FtsZ was detected in the absence of ZapE. Together, our results show that ZapE binds to PLs with ATP, and ZapE recruits FtsZ to the PL surface *in vitro*; however, neither interaction with PLs or FtsZ modifies ZapE ATP hydrolysis activity under the conditions tested.

### Deletion of *zapE* Impairs Division and Leads to a Synthetic Slow Growth Phenotype in a *minC* Deletion Strain

**FIGURE 4:**
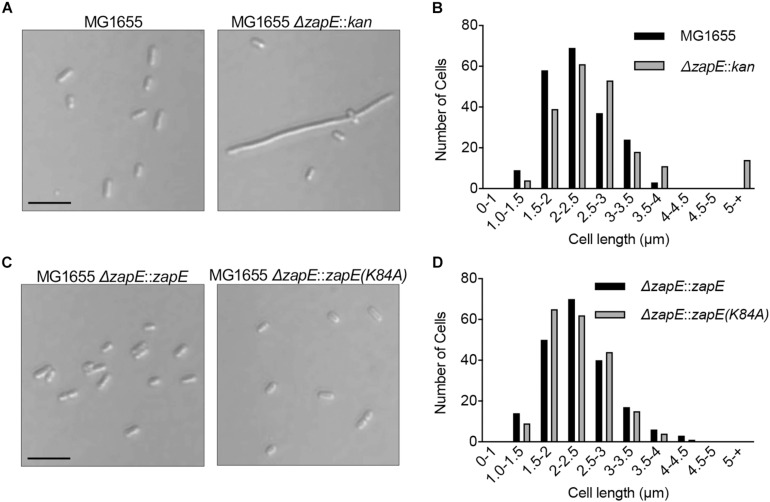
Cell morphology of *E. coli* strains deleted for *zapE.* **(A)** Log phase cultures of MG1655, MG1655 Δ*zapE* grown at 35°C in LB. Cells were collected and added to poly-_*L*_-lysine-coated coverslips and observed by DIC microscopy. Size bar is 5 μm. **(B)** Cell length distribution of MG1655 and MG1655 Δ*zapE* from morphology experiment in **(A)** (*n* = 200 cells). **(C)** Expression of ZapE wild type or mutant protein was restored in MG1655 Δ*zapE* with wild type *zapE* or *zapE(K84A)* at the native locus in the chromosome by recombination. Log phase cultures of MG1655 Δ*zapE*:*zapE* and MG1655 Δ*zapE*:*zapE(K84A)* were grown at 35°C in LB. Cells were collected and added to poly-_*L*_-lysine-coated coverslips and visualized by DIC microscopy. Size bar is 5 μm. **(D)** Cell length distribution of MG1655 Δ*zapE*:*zapE* and MG1655 Δ*zapE*:*zapE(K84A)* from morphology experiment in **(C)** (*n* = 200 cells).

ZapE was reported to be an ATPase and also a cell division protein in *E. coli* that is important for division under low-oxygen conditions and in cultures grown at high temperature (42°C) (Marteyn et al., 2014). First, to determine if we observe a phenotypic change in morphology following deletion of *zapE*, we constructed a *zapE* deletion strain in the *E. coli* K-12 strain MG1655 by Lambda Red recombination. Wild-type MG1655 cells and MG1655 Δ*zapE* cells were cultured in liquid LB medium under aerobic conditions, grown to log phase for 3 h at 35°C, and analyzed by microscopy to observe any morphologic changes. Both strains grew to a similar OD_600_ of approximately 0.8 A.U. As expected, *E. coli* MG1655 cells were short rods, with a mean length of 2.78 ± 0.53 μm (*n* = 200); however, cultures of MG1655 Δ*zapE* had a mean cell length of 3.86 ± 6.15 μm (*n* = 200), which is 38.8% longer than wild-type cells (Figures 4A,B), with a small population (7%) of filamentous cells longer than 5 μm. These results show that a moderate division phenotype is observed when cells have been deleted for *zapE* and grown aerobically at 35°C (Figure 4A). Notably, a mild filamentous phenotype was also previously shown for MG1655 Δ*zapE* cultured under aerobic conditions, and this phenotype was further exacerbated by anaerobiosis and elevated temperature (Marteyn et al., 2014). Next, to determine if ZapE ATP hydrolysis is required to support normal division, we constructed a strain expressing ZapE(K84A) from the native locus on the chromosome by replacing a *parE-kan* cassette at the original locus with *zapE*, to restore the original genotype or *zapE(K84A).* We performed microscopy on cells in log phase and determined that both strains, *zapE* + (restored) and *zapE(K84A)*, had a cell length distribution similar to wild-type MG1655 cells, and no filamentous cells were observed (Figures 4C,D). Additionally, MG1655 Δ*zapE*:*zapE* (restored) cells were observed to have a mean length of 2.26 ± 0.55 μm (n = 200), and MG1655 Δ*zapE*:*zapE(K84A)* cells were observed to have a mean length of 2.31 ± 0.60 μm (*n* = 200) (Figure 4D). These results suggest that deletion of *zapE* leads to a mild cell division defect in *E. coli* and that ATP hydrolysis does not appear to be critical for this function, since *zapE(K84A)* restores the moderate filamentation defect, similar to *zapE*.

**FIGURE 5:**
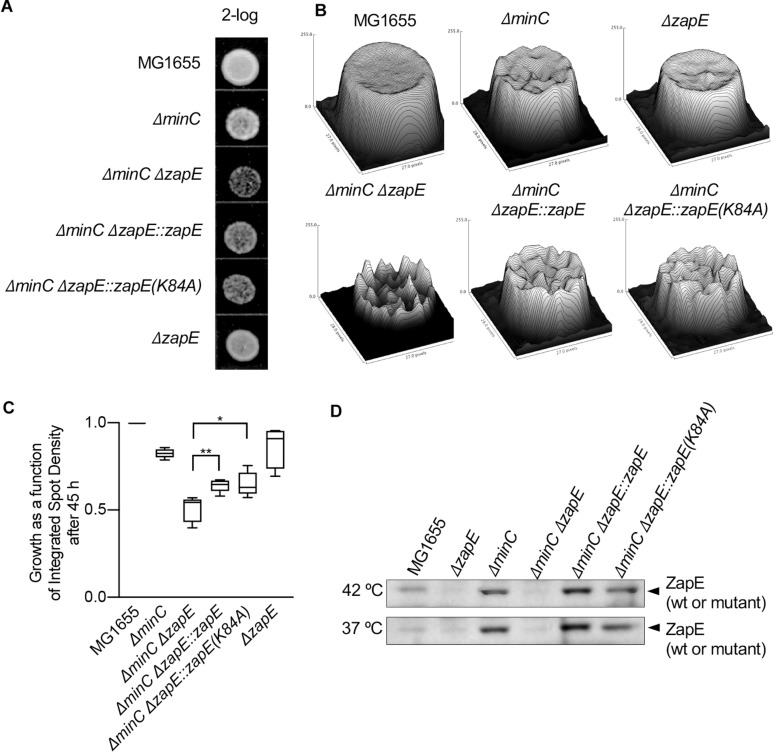
Cells deleted for *zapE* and *minC* grow slowly after exposure to thermal and oxygen stress. **(A)** Overnight cultures MG1655, MG1655 Δ*zapE*, MG1655 Δ*minC*, MG1655 Δ*minC*Δ*zapE*:*kan*, MG1655 Δ*minC*Δ*zapE*:*zapE(K84A)*, and MG1655 Δ*minC*Δ*zapE*:*zapE* were diluted in to fresh medium (LB broth) and exposed to thermal stress (42°C) and low oxygen conditions for 3 h. Then, dilutions (2-log) were spotted (5 μl) on no salt LB agar and incubated at room temperature. **(B)** Images of spots were collected and analyzed by surface mapping in ImageJ to compare biomass and visualized in a three-dimensional plot of density versus pixel area. Biomass was evaluated by comparing integrated spot density after 45 h of growth. **(C)** Growth analysis in **(B)** was collected across five replicate experiments. Average biomass as a function of integrated spot density is represented as a box and whiskers plot. (p-values are as follows: *<0.05 and **<0.01). **(D)** Overnight cultures MG1655, MG1655 Δ*zapE*, MG1655 Δ*minC*, MG1655 Δ*minC*Δ*zapE*:*kan*, MG1655 Δ*minC*Δ*zapE*:*zapE(K84A)*, and MG1655 Δ*minC*Δ*zapE*:*zapE* were diluted into fresh medium (LB broth) and exposed to thermal stress (42°C) and low oxygen conditions, or at 37°C and shaking, for 3 h. Cells were harvested and analyzed for ZapE expression by immunoblotting.

The cell division defect detected in the deletion strain is moderate, therefore we tested if cultures exposed to high temperature and/or oxygen stress developed a more severe growth defect. First, we cultured wild type MG1655 cells and MG1655 Δ*zapE* cells on solid LB medium without O_2_ by dilution spot-plating; however, we observed no major differences in the extent of growth of the two strains after 24 h (Supplementary Figure 3A). Then, we tested if exposure to both high temperature and oxygen stress exacerbated any growth defects. We incubated the cells at 42°C for 3 h in static cultures with no air contact to induce thermal and oxygen stress, then spotted culture dilutions onto LB agar plates, and incubated the plates at 23°C (Figure 5A). We observed that cultures of cells exposed to stress grew slower than cells cultured aerobically at 35°C after 3 h (Supplementary Figures 3B,C); however, this was independent of the absence or presence of *zapE.* We then spot plated the cultures after exposure to stress and observed that both wild type and *zapE* deletion cells grew to a similar extent on plates after the initial exposure to stress (Figures 5A–C and Supplementary Figure 3D). Moreover, the morphologies of stressed *zapE* deletion cells also appeared similar to morphologies of unstressed cells cultured aerobically at 30°C [2.19 ± 0.15 μm (*n* = 100) and 2.32 ± 0.12 μm (*n* = 100), respectively], with occasional cell filaments observed under both conditions (3% and 2%, respectively) (Supplementary Figures 3E,F).

Next, to determine if the deletion of *zapE* in combination with chromosomal deletion of another FtsZ assembly regulator, such as *minC*, further altered cell growth or morphology, we constructed a strain deleted for both *minC* and *zapE* by Lambda Red recombination. It was previously reported that deletion of either *slmA* or *clpX* caused a synthetic phenotype in combination with deletion of *minC* (Bernhardt and de Boer, 2005; Camberg et al., 2011) and that the *minC* deletion strain may be more sensitive to deletion of non-essential FtsZ assembly regulators. MinC is a cell division protein that functions in the cell to prevent assembly of the Z-ring near the cell poles by destabilizing FtsZ polymers (Hu et al., 1999; Hu and Lutkenhaus, 2000; Dajkovic et al., 2008; LaBreck et al., 2019). To evaluate the synthetic phenotypes of *minC* and *zapE* deletion strains, we first compared morphologies, since deletion of *minC* alone induces mild filamentation and minicell formation. We observed that cultures of cells deleted for both *minC* and *zapE* contained short filaments and minicells, similar to the cells deleted for *minC* alone, even after culturing cells at high temperature with oxygen stress (Supplementary Figures 3E,F). However, following exposure to stress, we observed that cells deleted for both *minC* and *zapE* recovered poorly, in contrast to the cells deleted for either *minC* or *zapE.* Analysis of cultured spot densities showed severe growth delays that were approximately 50% slower than wild type MG1655 cells (Figures 5A–C). Reinsertion of *zapE* or *zapE(K84A)* at the chromosomal *zapE* locus restored the ability to recover after exposure to stress (Figure 5C). These results show that after exposure to stress, the cells deleted for *zapE* and *minC* are less able to resume normal growth and experience a growth delay on solid LB agar. Finally, we tested if exposure to stress induces expression of *zapE* in wild type cells. We cultured wild type, *minC* and *zapE* deletion strains under stressed (42°C, oxygen stress) and non-stressed conditions (37°C, aerobic with shaking). We observed that ZapE was present in wild-type cells under both stressed and non-stressed conditions and not present in Δ*zapE* or Δ*zapE*Δ*minC* deletion strains (Figure 5D). Interestingly, ZapE appears to be present at higher levels in the cells deleted for *minC* in both stressed and non-stressed cells (Figure 5D); however, it is unclear if this is due to transcriptional regulation or modified post-translational degradation. Together, these results show that although ZapE is important for resuming division following stress, it does not appear that it is induced or synthesized to a large extent in response to exposure to environmental stress.

## Discussion

Here, we report that ZapE is an ATPase that binds directly to FtsZ and bundles GTP-induced FtsZ polymers in light scattering assays and by TEM, and this interaction requires ATP. We also show that ZapE binds to PL vesicles *in vitro* with ATP and can recruit the chimeric protein Gfp-FtsZ to PL vesicles. While FtsZ polymer bundling activity was observed *in vitro* in the absence of PLs, it is unclear if the bundling activity also occurs while ZapE is bound to PLs. To date, only two other proteins in *E. coli* have been reported to localize FtsZ to the PL membrane, FtsA, and ZipA (Hale and de Boer, 1997, 1999; Liu et al., 1999; Hale et al., 2000; Haney et al., 2001; Pichoff and Lutkenhaus, 2002; Conti et al., 2018). Although ZapE is not essential for survival, it is unclear if ZapE shares any redundant functions or overlapping roles with either FtsA or ZipA (Pichoff and Lutkenhaus, 2002; Geissler et al., 2003). The interaction between ZapE and PLs is likely electrostatic, since it is disrupted by high salt concentration; however, the interaction is tolerant to 400 mM NaCl, so it is likely relevant *in vivo* (Supplementary Figure 2H). Accordingly, ZapE was previously reported in association with the bacterial inner membrane by EM (Marteyn et al., 2014). As ATP is required for ZapE to bundle FtsZ polymers in light scatter assays and to bind to PLs ***in vitro*** (Figures 2C, 3A), this suggests that a potential role for ZapE may be to stabilize FtsZ polymer assemblies adjacent to the membrane. Furthermore, this function appears to be important for ensuring growth during and after exposure to stress.

ZapE is member of the AAA + superfamily of ATPases and, in our study, we show that ZapE is a monomer with and without ATP (Supplementary Figures 1B,C). ZapE shares sequence homology with other AAA + ATPases, including DnaA, DnaC, FtsH, and VCP/p97 (Peters et al., 1992; Akiyama et al., 1995; Tomoyasu et al., 1995; Karata et al., 2001; Davies et al., 2008; Duderstadt and Berger, 2008; Mogk et al., 2008). AAA + ATPases have two fundamental regions important for ATP binding and subsequent hydrolysis, Walker A and Walker B motifs. The Walker A Lys (K84) residue is crucial for ATP hydrolysis of ZapE *in vitro* since we observed that ZapE(K84A) is defective for ATP hydrolysis; however, ZapE(K84A) binds to and bundles FtsZ polymers. Many AAA + ATPases (i.e., ClpX) form hexameric rings, and may be stabilized when bound to nucleotide (Grimaud et al., 1998) (i.e., FtsH and VCP/p97) (Akiyama et al., 1995; Tomoyasu et al., 1995; Karata et al., 2001; Davies et al., 2008; Duderstadt and Berger, 2008; Mogk et al., 2008). Although less common, there are examples of stable monomeric AAA + proteins, including the initiator of DNA replication, DnaA, and the eukaryotic cell division, and DNA replication regulator Cdc6 (cell division control protein 6) (Liu et al., 2000; Rozgaja et al., 2011). The mechanism of FtsZ polymer bundling by ZapE is still unclear; however, if ZapE functions as a monomer during the bundling activity, then it may contain two FtsZ-interaction sites per ZapE protomer that could bridge two protofilaments. Other Zaps (A–D) also interact with FtsZ and influence bundling of FtsZ protofilaments, although the Zaps are not structurally related. ZapA interacts directly with FtsZ, while ZapB does not, but is recruited to midcell by ZapA and enhances the activity of ZapA to promote FtsZ filament bundling (Low et al., 2004; Small et al., 2007; Galli and Gerdes, 2012; Buss et al., 2013; Roach et al., 2014; Caldas et al., 2019). *In vitro*, ZapA forms a tetramer and cross-links FtsZ polymers, while reducing the GTPase activity of FtsZ (Gueiros-Filho and Losick, 2002; Low et al., 2004; Small et al., 2007; Pacheco-Gomez et al., 2013). ZapC also promotes the bundling of FtsZ polymers and inhibits FtsZ GTP hydrolysis (Hale et al., 2011). Deletion of *zapC* leads to a minor cell elongation phenotype, which is exacerbated by additional deletions in *zapA* or *zapB* and further suggests a role for ZapC in Z-ring stability (Hale et al., 2011; Durand-Heredia et al., 2012). *In vitro*, ZapD cross-links FtsZ polymers and reduces the GTPase activity of FtsZ (Durand-Heredia et al., 2012). Deletion of *zapD* alone confers no defect in viability; however, in combination with temperature-sensitive mutation in the *ftsZ* gene, *ftsZ*(G105S), deletion of *zapD* leads to filamentation and a decrease in viability, suggesting the role of ZapD as a regulator of FtsZ (Durand-Heredia et al., 2012). While ZapA, ZapC, and ZapD all bundle FtsZ, leading toward a reduction in the rate of FtsZ in GTP hydrolysis, we detected no decrease in FtsZ GTP hydrolysis activity in the presence of ZapE under the conditions tested, which may suggest a novel mechanism for FtsZ polymer bundling or crosslinking. Moreover, the previous characterization of ZapE reported that ZapE disassembles FtsZ polymers in reactions containing Ca^2+^, which is known to support the formation of bundled sheets of FtsZ and GTP (Yu and Margolin, 1997; Lowe and Amos, 1999; Marteyn et al., 2014). We did not detect FtsZ protofilament disassembly activity by sedimentation, light scatter, or TEM under the conditions tested. However, we did detect that ZapE binds to unassembled FtsZ and suspect that under some conditions, ZapE could sequester FtsZ monomers and prevent them from participating in polymerization, or that ZapE modulates Ca^2+^-dependent sheet formation of FtsZ in the presence of GTP. Our work shows that the FtsZ polymer bundling function of ZapE is calcium independent (Figures 2A,E). Additional work will be necessary to determine if ZapE performs a holdase or destabilizing function that modifies FtsZ polymer dynamics *in vivo*. A previous report demonstrated that the exchange of Gfp–FtsZ polymer subunits within the Z-ring in dividing cells is slower in cells deleted for *zapE*, indicating that ZapE influences FtsZ structures at the division site and supporting the model that ZapE contributes to the regulation of FtsZ polymer assemblies during division (Viola et al., 2017).

In *E. coli*, deletion of *zapE* was previously reported to produce an elongated cell phenotype exacerbated by anaerobic conditions or elevated temperature (42°C) (Marteyn et al., 2014). Our study also demonstrated conditions in which deletion of *zapE* generated a subpopulation of filamentous cells under the conditions tested. We also observed a synthetic slow growth phenotype in cells deleted for *minC* and *zapE* after exposure to mild heat shock, which is more severe than deletion of either gene individually. Synthetic phenotypes have also been observed in other strains when *minC* was deleted in combination with *slmA* (synthetic lethal) or *clpX* (synthetic filamentous) (Bernhardt and de Boer, 2005; Camberg et al., 2011). MinC spatially regulates placement of the Z-ring *in vivo* by preventing Z-ring assembly near the cell poles (Hu et al., 1999; Hu and Lutkenhaus, 2000; Dajkovic et al., 2008; LaBreck et al., 2019). In cells deleted for *minC*, ZapE may function to organize FtsZ near the membrane to support future rounds of proliferation. Our results establish that when division site selection is impaired by deletion of *minC*, or when cells are under stress, other FtsZ assembly regulators including ZapE are important for promoting division.

## Data Availability Statement

The original contributions presented in the study are included in the article/Supplementary Material, further inquiries can be directed to the corresponding author.

## Author Contributions

ED, RD, CT, and JC contributed to conception and design of the study. ED, RD, CT, and CF contributed to assay development and performed experiments. JM performed electron microscopy. ED wrote the first draft of the manuscript. CT, CF, JM, and JC wrote sections of the manuscript. All authors contributed to manuscript revision, read, and approved the submitted version.

## Conflict of Interest

The authors declare that the research was conducted in the absence of any commercial or financial relationships that could be construed as a potential conflict of interest.

## Publisher’s Note

All claims expressed in this article are solely those of the authors and do not necessarily represent those of their affiliated organizations, or those of the publisher, the editors and the reviewers. Any product that may be evaluated in this article, or claim that may be made by its manufacturer, is not guaranteed or endorsed by the publisher.
