# Supplementary material for: The Stress-Active Cell Division Protein ZapE Alters FtsZ Filament Architecture to Facilitate Division in Escherichia coli
Source: Front Microbiol. 2021 Sep 27;12:733085. doi: 10.3389/fmicb.2021.733085 (PMC8503651; doi:10.3389/fmicb.2021.733085)
Supplement: Supplementary file 1 [file Data_Sheet_1.PDF]

Supporting information for: "The stress-active cell division protein ZapE alters FtsZ filament architecture to facilitate division"

Eric C. DiBiasio[1], Rebecca A. Dickinson[1], Catherine E. Trebino[1], Colby N. Ferreira[1], Josiah J. Morrison[1], Jodi L. Camberg[1#]

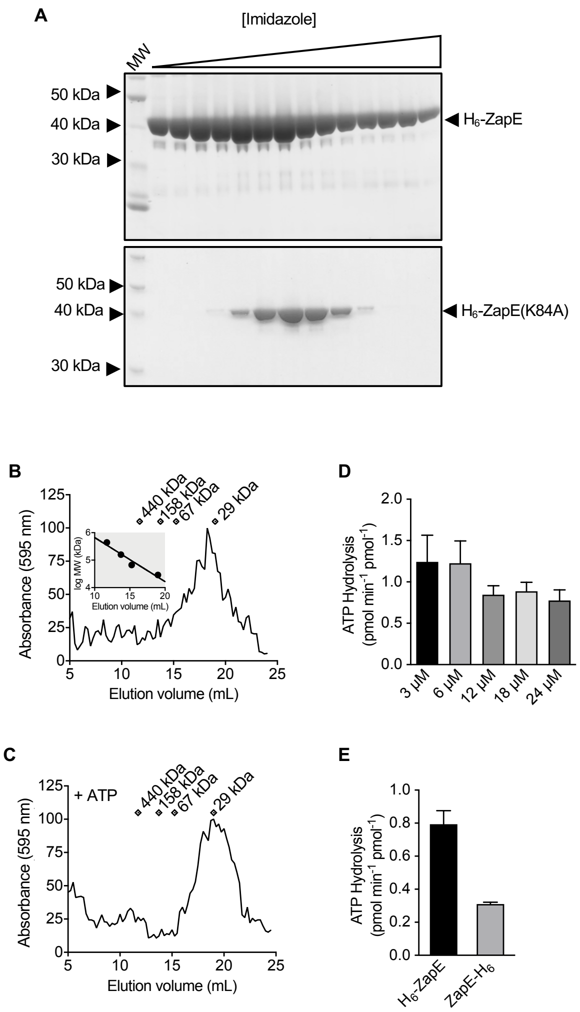

Supplemental Figure S1 – ZapE hydrolyzes ATP.

(A) His_6-ZapE and His_6-ZapE(K84A) were purified by metal affinity chromatography as described in Material and Methods and analyzed by SDS-PAGE.

(B) The elution profile of His_6-ZapE was collected by size exclusion chromatography where each 0.25 mL fraction (5 – 25 ml) was tested for protein concentration represented by intensity (A.U.), at a wavelength of 595 nm, using Bradford reagent detection. Protein standards ferritin, aldolase, bovine serum albumin, and carbonic anhydrase (GE Healthcare) were used to calibrate the column and plotted as log molecular weight (MW) versus elution volume (inset).

(C) His_6-ZapE was analyzed as in (B) in the presence of ATP.

(D) Hydrolysis of ATP (4 mM) by across a range of ZapE concentrations (0, 3.0, 6.0, 12.0, 18.0, and 24.0 µM) was measured as described *Materials and Methods*.

(E) Hydrolysis of H_6-ZapE (12 µM) and ZapE-H_6 (12 µM) represented by pmol Pi min^{-1} $pmol^{-1}$. Data shown is an average of at least three replicates represented as mean ± SEM.

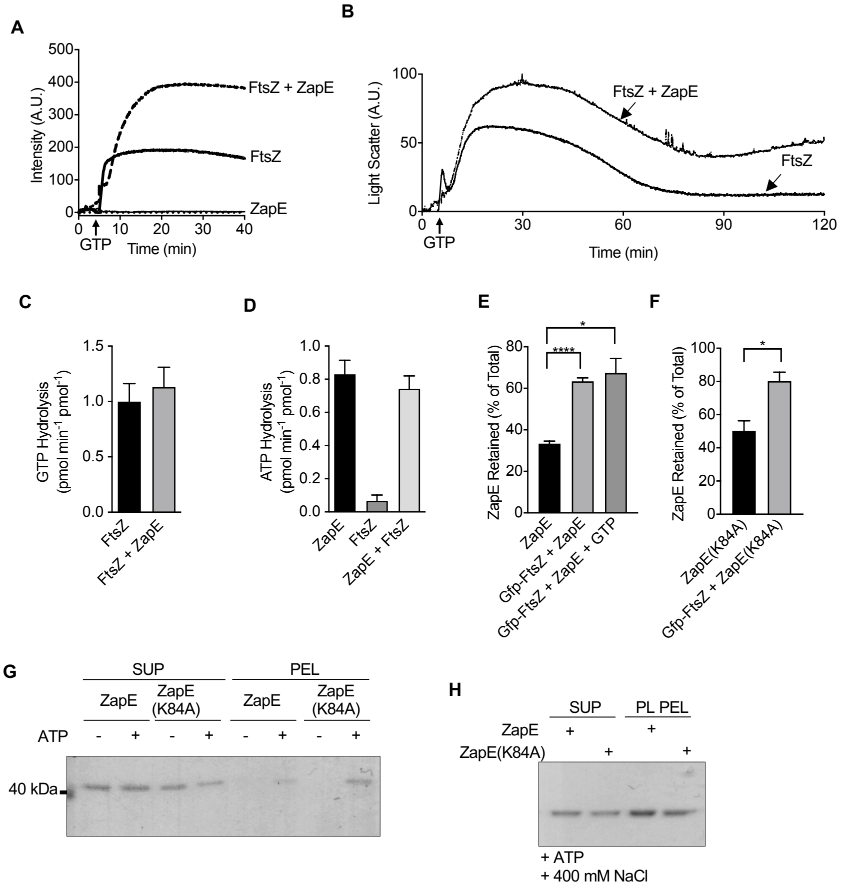

Supplemental Figure S2 – Biochemical assays characterizing the direct interaction between ZapE and FtsZ.

(A) Reactions containing ZapE (8 μM), FtsZ (8 μM), or FtsZ (8 μM) and ZapE (8 μM), were incubated for 5 min with ATP (4 mM), and then GTP (1.5 mM) was added. Reactions were monitored by 90° light scattering for 40 min as described in *Materials and Methods*.

(B) Reactions containing FtsZ (8 μM) alone or with ZapE (8 μM) and ATP were incubated for 5 min, and then GTP (1.5 mM) was added. Reactions were monitored by 90° light scattering for 120 min to monitor complexes in an extended assay.

(C) GTP (1.5 mM) hydrolysis assay of FtsZ (6 μM) alone or with ZapE (12 μM), represented by pmol Pi min^{-1} $pmol^{-1}$.

(D) ATP hydrolysis assay of ZapE (12 μM), FtsZ (6 μM), or ZapE (12 μM) and FtsZ (6 μM), with ATP (4 mM), represented by pmol Pi min^{-1} $pmol^{-1}$.

(E) Protein filter retention assays were performed by incubated ZapE (5 μM) alone or with and/or Gfp-FtsZ (10 μM), ATP (4 mM), and GTP (2 mM), where indicated. Complexes were collected by ultrafiltration, analyzed by SDS-PAGE and densitometry. Data shown is an average of at least three replicates represented as mean ± SEM.

(F) Protein filter retention assays were performed by incubated ZapE(K84A) (5 μM) as described in (E). Data shown is an average of at least three replicates represented as mean ± SEM.

(G) ZapE (1 μM) or, where indicated, ZapE(K84A) (1 μM), was incubated without *E. coli* PL vesicles in the absence or presence of ATP (4 mM). Pellet-associated protein was collected centrifugation. Supernatants and pellets were analyzed by SDS-PAGE. Data shown is representative of at least three replicates.

(H) ZapE (1 μM) or, where indicated, ZapE(K84A) (1 μM), was incubated with *E. coli* PL vesicles (500 μg ml^{-1}) in the absence or presence of ATP (4 mM) and 400 mM KCl to disrupt electrostatic interactions. PL-associated protein was collected by low speed centrifugation. Supernatants and pellets were analyzed by SDS-PAGE.

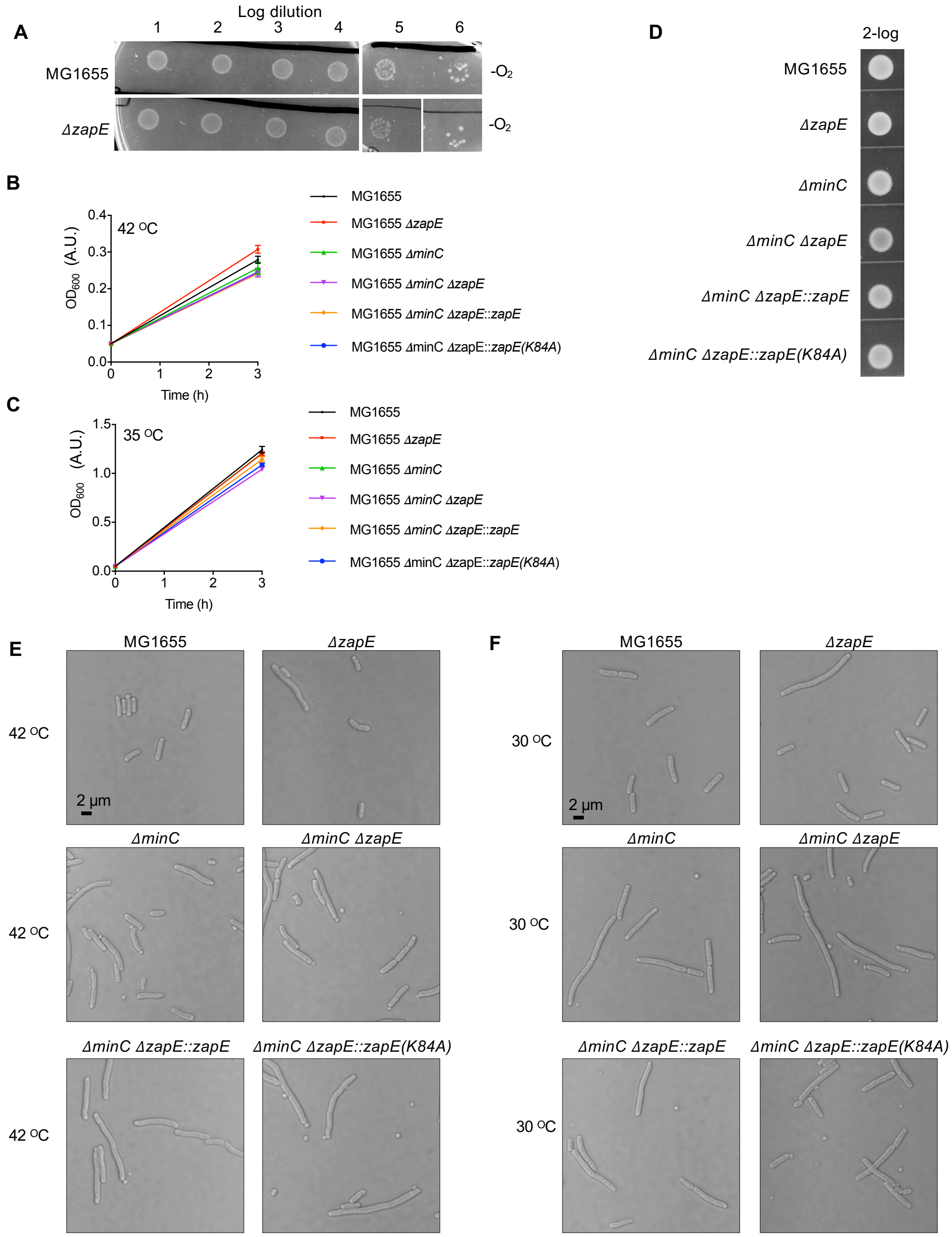

Supplemental Figure S3 – Growth of wild type and *zapE* deletion strains**.**

(A) Overnight cultures of MG1655 and MG1655 *ΔzapE* were diluted and grown in static air-free cultures at 37 °C for 3 h, and then log dilutions were spotted on LB agar plates. Plates were incubated at 37 °C in an anaerobic GasPak (BD Biosciences) chamber for 24 h.

(B) Overnight cultures of MG1655, MG1655 *ΔzapE,* MG1655 *ΔminC,* MG1655 *ΔminC ΔzapE,* MG1655 *ΔminC ΔzapE*::*zapE(K84A)* and MG1655 *ΔminC ΔzapE*::*zapE* were diluted into fresh no salt LB medium and incubated at 42 °C in static, air-free cultures. After 3 h, growth was measured by OD_{600}. Data from three replicates are shown as mean ± SEM.

(C) Overnight cultures of MG1655, MG1655 *ΔzapE,* MG1655 *ΔminC,* MG1655 *ΔminC ΔzapE,* MG1655 *ΔminC ΔzapE*::*zapE(K84A)* and MG1655 *ΔminC ΔzapE*::*zapE* were diluted into fresh no salt LB medium and incubated at 35 °C, shaking. After 3 h, growth was measured by OD_{600}. Data from three replicates are shown as mean ± SEM.

(D) Cultures in (B) were diluted (2-log) and spotted (5 µl) onto LB agar and monitored for colony development at 23 °C. Size bar is 2 µm.

(E) Cells in (A) were analyzed by DIC microscopy after exposure to stress for 3 h.

(F) Cultures of MG1655, MG1655 *ΔzapE,* MG1655 *ΔminC,* MG1655 *ΔminC ΔzapE,* MG1655 *ΔminC ΔzapE*::*zapE(K84A)* and MG1655 *ΔminC ΔzapE*::*zapE* were grown to log phase at 30 °C and analyzed by DIC microscopy. Size bar is 2 µm.
